# Performance of Multiple-Batch Approaches to Pharmacokinetic Bioequivalence Testing for Orally Inhaled Drug Products with Batch-to-Batch Variability

**DOI:** 10.1208/s12249-021-02063-1

**Published:** 2021-08-19

**Authors:** Elise Burmeister Getz, Kevin J. Carroll, J. David Christopher, Beth Morgan, Scott Haughie, Alessandro Cavecchi, Christopher Wiggenhorn, Hayden Beresford, Helen Strickland, Svetlana Lyapustina

**Affiliations:** 1 Clinical Pharmacology, Sandoz Inc., 5959 Horton Street, Emeryville, California, 94608 USA; 2 KJC Statistics Limited, Cheshire, UK; 3 Merck & Co., Inc., West Point, Pennsylvania USA; 4 Inhaled Product Development, AstraZeneca, Durham, North Carolina USA; 5 Biometrics-Global Clinical Operations, Viatris/Mylan Pharma UK Ltd., Sandwich, Kent, UK; 6 CMC-Quality Control & Product Development, Chiesi Farmaceutici S.p.A., Parma, Italy; 7 Research & Development, Kindeva Drug Delivery, St Paul, Minnesota USA; 8 Formulation Development, Kindeva Drug Delivery, Loughborough, UK; 9 Manufacturing Science & Engineering, GlaxoSmithKline, Zebulon, North Carolina USA; 10 Pharmaceutical Consortia Management Team, Faegre Drinker Biddle & Reath LLP, Washington, DC USA

**Keywords:** bioequivalence, pharmacokinetic, batch-to-batch variability, multiple-batch, type I error

## Abstract

Batch-to-batch pharmacokinetic (PK) variability of orally inhaled drug products has been documented and can render single-batch PK bioequivalence (BE) studies unreliable; results from one batch may not be consistent with a repeated study using a different batch, yet the goal of PK BE is to deliver a product comparison that is interpretable beyond the specific batches used in the study. We characterized four multiple-batch PK BE approaches to improve outcome reliability without increasing the number of clinical study participants. Three approaches include multiple batches directly in the PK BE study with batch identity either excluded from the statistical model (“Superbatch”) or included as a fixed or random effect (“Fixed Batch Effect,” “Random Batch Effect”). A fourth approach uses a bio-predictive *in vitro* test to screen candidate batches, bringing the median batch of each product into the PK BE study (“Targeted Batch”). Three of these approaches (Fixed Batch Effect, Superbatch, Targeted Batch) continue the single-batch PK BE convention in which uncertainty in the Test/Reference ratio estimate due to batch sampling is omitted from the Test/Reference confidence interval. All three of these approaches provided higher power to correctly identify true bioequivalence than the standard single-batch approach with no increase in clinical burden. False equivalence (type I) error was inflated above the expected 5% level, but multiple batches controlled type I error better than a single batch. The Random Batch Effect approach restored 5% type I error, but had low power for small (*e.g.*, <8) batch sample sizes using standard [0.8000, 1.2500] bioequivalence limits.

**Supplementary Information:**

The online version contains supplementary material available at 10.1208/s12249-021-02063-1.

## INTRODUCTION

Sampling variability is an important consideration in clinical study design and interpretation. Investigators infer a conclusion about a population, *e.g.*, the effect of a new medicine, from data collected on a sample of the population. Different samples will give different results, yet only one sample of the population is observed in any given study. Investigators account for sampling variability when drawing conclusions based on the observed sample, and often reduce sampling error by increasing the size of the sample.

Sample size calculations are routinely performed to estimate the number of clinical study subjects required for a study, but consideration is rarely given to the number of drug product samples (*i.e.*, manufacturing batches) to be used and, in particular, whether variability between product samples is an additional, separate source of sampling variability. For many drug products, this may not be a problem because of negligible *in vivo* variability from batch to batch. However, substantial variability in pharmacokinetic (PK) response (*e.g.*, maximum blood concentration (Cmax), total blood exposure to the drug (AUC)) between batches of orally inhaled drug products (OIDPs) is well established. This is perhaps not surprising; the low systemic availability of OIDPs creates a wide window of opportunity for PK variation, and the complex nature of OIDP process parameters and quality attributes limits control of *in vivo* performance. Yet the standard pharmacokinetic bioequivalence (PK BE) study, even for OIDPs, uses only a single batch of each product. When batches of a product are known to differ, the use of only one to draw inference about the product population can lead to decision errors. The confounding influence of batch-to-batch PK variability on PK BE decision-making for OIDPs has been a topic of discussion among pharmaceutical industry scientists and regulators for over a decade (1–3).

The impact of batch-to-batch PK variability on bioequivalence testing is most clearly illustrated by comparing a marketed product to itself across batches using standard study design and analysis methods. For example, Advair Diskus® has repeatedly failed to pass the standard PK BE test when compared to itself because PK differences between batches were either too large to pass PK BE in an appropriately powered study (4), or were so large as to demonstrate PK bio-*in*equivalence (5) of one batch to another (6). In these cases, truly equivalent treatments, *i.e.*, different batches of the same product, erroneously fail the PK BE test. Similarly, between-batch PK variability can cause truly non-equivalent treatments to incorrectly pass PK BE. Both decision errors are a consequence of using an inadequate batch sample size (one, in the standard PK BE study) to infer a conclusion about a variable product population. Incorporating multiple batches into the PK BE assessment may mitigate this uncertainty in PK BE outcome.

The International Pharmaceutical Aerosol Consortium on Regulation and Science (IPAC-RS) convened a working group to characterize the performance of various multiple-batch PK BE study design and data analysis approaches. The work aimed to quantitatively assess the impact of increasing batch sample size (number of batches) without increasing the number of clinical study participants. In total, four multiple-batch approaches were characterized and their quantitative performance presented alongside that of the standard single-batch crossover. The multiple-batch approaches are summarized as follows.

### Random Batch Effect

Multiple batches are used in the PK BE study, with batch included as a random factor in the statistical analysis of variance (ANOVA) model. Thus, batch sampling is explicitly acknowledged as an additional source of sampling variability contributing to the uncertainty of the estimated Test/Reference (T/R) ratio. This approach allows the observed PK BE result to be generalized beyond the selected batches; the T/R ratio confidence interval recognizes that the specific PK BE batches and measurements are simply samples from variable populations. The Random Batch Effect approach implicitly asks if the Test *product* is PK BE to the Reference *product*, instead of asking if the selected Test *batches* are PK BE to the selected Reference *batches*.

### Fixed Batch Effect

Multiple batches are used in the PK BE study, with batch included as a fixed factor in the ANOVA; batch is not considered an additional source of sampling variability. The resulting T/R ratio’s 90% confidence interval therefore requires careful interpretation; expected coverage properties, *i.e.*, that 90% of such intervals from repeated trials contain the true T/R value, are valid only for the selected batches. The Fixed Batch Effect approach implicitly asks if the selected Test *batches* are PK BE to the selected Reference *batches*; it does not ask if the *products* are PK BE.

### Superbatch

Multiple batches are used in the PK BE study, with batch identity omitted from the ANOVA; the composite data from several batches appears to the ANOVA as a single “superbatch” of Test or Reference. PK variability between the selected batches is subsumed into residual error. This approach was originally proposed by Sandell in 2015 (*e.g.*, “Inhaled Drug Delivery, London, UK, November 19–20, 2015”) and later elaborated by Sandell and colleagues (7). The approach offers statistical simplicity because the conventional single-batch PK BE ANOVA model proceeds without modification; treatment is simply identified as either “Test” or “Reference” without additional model terms associated with batch identity. However, as with the preceding Fixed Batch Effect approach, the Superbatch T/R ratio’s confidence interval largely omits uncertainty due to batch sampling and is therefore correct only for the observed batches.

### Targeted Batch

*In vitro* (or other bio-predictive) data from multiple batches guide the selection of one batch of each product for the PK BE study, which is then conducted using conventional single-batch PK BE design and analysis methods. This approach has been suggested by, for example, the European Medicines Agency (EMA) Pharmacokinetics Working Party (8). For simplicity, the implementation of the Targeted Batch approach considered here selects the batch that sits at the predictive metric (*e.g.*, *in vitro*) median and assumes that the *in vitro* measurement is perfectly correlated with the PK endpoints (Cmax, AUC). The PK BE ANOVA does not take into account the batch selection process; as in the preceding Fixed Batch Effect and Superbatch approaches, uncertainty arising from batch sampling is omitted and, hence, again, the T/R ratio’s confidence interval is directly interpretable only in the context of repeated trials using the same originally observed batches.

## METHODS

### Study Design

Comparisons among the approaches are considered in the context of a single harmonized design: a two-period, single-group, randomized crossover. Multiple batches are incorporated into the *in vivo* study by arranging the study subjects into cohorts (Table I). Each cohort of subjects receives a single batch of Test (T) and a single batch of Reference (R) in random order (TR or RT) across the two treatment periods. Different cohorts receive different batches. The total number of batches is the same for Test and Reference in the designs considered here, although the principles remain relevant to designs with a different number of Test and Reference batches. The multiple-cohort design divides the total number of study subjects into *c* smaller 2×2 sub-studies (cohorts), then combines the results across cohorts *via* a single analysis of variance (ANOVA) model to estimate overall PK relative systemic availability (the T/R ratio). Throughout, *c* = number of cohorts = number of batches per product dosed in the PK BE study; *m* = number of subjects per sequence per cohort. The total number of study subjects (N) is therefore 2*mc* and the total number of observations per PK metric is 4*mc*.

**Table I:** Multiple-Cohort PK BE Example Study Design for Including Multiple Batches. *c* individual batches of Test (T1 through T*c*) and Reference (R1 through R*c*) are compared, with one T-*vs*-R batch pair observed in each of *c* cohorts

Cohort	Sequence	Treatment		# Subjects
		Period 1	Period 2	
1	1	T1	R1	*m*
	2	R1	T1	*m*
2	1	T2	R2	*m*
	2	R2	T2	*m*
….	….	….	….	
*c*	1	T*c*	R*c*	*m*
	2	R*c*	T*c*	*m*

### Statistical Model

Sources of variability and degrees of freedom (*df*) associated with each ANOVA model term (Table II) are identified as fixed or random by each multiple-batch approach as indicated in Table III. The statistical model is detailed in Supplement 1.

**Table II:** ANOVA Table for a Two-Period Multiple-Batch Bioequivalence Study Run in *c* Cohorts. Sources of variability and associated degrees of freedom (*df*) for a two-way crossover PK BE study that includes multiple batches by grouping subjects into cohorts (Table I). *m*, number of subjects per sequence per cohort; *c*, number of cohorts = number of batches per product

Source	*df*	
*Cohort*	*c* − 1	*Between-subject effects*
*Seq*	1	
*Seq* × *Cohort*	*c* − 1	
*Subj*(*Seq* × *Cohort*)	2*c*(*m* − 1)	
*Treat* × *Cohort*^*1*^	*c* − 1	*Within-subject effects*
*Period*	1	
*Treat*	1	
*Error*^*2*^	2*mc* − *c* − 1	
*Total*	4*mc* − 1	

^*1*^The Treatment-by-Cohort interaction term (*Treat* × *Cohort*) has zero degrees of freedom for designs that use single batches of Test and Reference in the PK BE study (*c*=1), and therefore does not appear in the ANOVA model for these designs. (*Treat* × *Cohort*) is also omitted in the Superbatch approach, effectively instructing the model to consider that all data come from a single “superbatch” each of Test and Reference

^*2*^Approaches that omit (*Treat* × *Cohort*) have an Error degrees of freedom that is increased by *c*−1, *i.e.*, Error *df* = 2*mc*−2

**Table III:** Model-Dependent Standard Error of the Log(T/R) Estimate. Random Model Terms Are Distinguished from Fixed Effects with Italics and Bold. *Seq*, treatment sequence; *Subj*, clinical study subject; *Treat*, treatment (test, reference); $\overset{\sim }{SE}$, $\overset{\sim }{df}$, model-specific standard error and degrees of freedom (See Supplement 2); *MS*, mean square; ${\sigma}_b^2$, log-scale within-subject, between-batch PK variance, assumed equal for test and reference; ${\sigma}_e^2$, log-scale within-subject residual error PK variance; *m*, number of subjects per sequence per cohort; *c*, number of cohorts = number of batches per product

Approach	Terms in the statistical model	Model-dependent values implemented in construction of the log(T/R) confidence interval		
		True value of the model-defined $\overset{\sim }{SE}$	ANOVA component used to estimate $\overset{\sim }{SE}$	$\overset{\sim }{df}$
Random Batch Effect	Cohort Seq Seq × Cohort ***Subj(Seq*** × ***Cohort)*** Treat ***Treat*** × ***Cohort*** Period ***Error***	$\sqrt{\frac{\sigma_e^2}{mc}+\frac{2{\sigma}_b^2}{c}}$	$\sqrt{\frac{MS\left(T\ast C\right)}{mc}}$	*c* – 1
Fixed Batch Effect	Cohort Seq Seq × Cohort ***Subj(Seq*** × ***Cohort)*** Treat Treat × Cohort Period ***Error***	$\sqrt{\frac{\sigma_e^2}{mc}}$	$\sqrt{\frac{MS(Error)}{mc}}$	2*mc* – *c* – 1
Superbatch	Seq ***Subj(Seq)*** Treat Period ***Error***	$\sqrt{\frac{\sigma_e^2+2{\sigma}_b^2\left(\frac{m\left(c-1\right)}{\left(2 mc-2\right)}\right)}{mc}}$	$\sqrt{\frac{MS(Error)}{mc}}$	2*mc* – 2
Targeted Batch	Seq ***Subj(Seq)*** Treat Period ***Error***	$\sqrt{\frac{\sigma_e^2}{m}}$	$\sqrt{\frac{MS(Error)}{mc}}$	2*m* – 2

### Variance (and Standard Error) of the Estimated Log(Test/Reference) Ratio

The approach-specific standard error (SE) and *df* of the log(T/R) estimate (Table III) illustrate that only the Random Batch Effect approach uses the correct log(T/R) confidence interval in the bioequivalence test, given random batch variability; all other approaches construct a log(T/R) confidence interval from SE and *df* values that do not describe the true variability of the treatment difference population from which the data sample was drawn. The SE of the estimated log(T/R) ratio implemented by each multiple-batch approach is summarized as follows.

#### Random Batch Effect

When (*Treat* × *Cohort*) is handled as a random effect, the Treatment term has an expected mean square (MS) value of ${\sigma}_e^2+2m{\sigma}_b^2+f\left({\theta}_i\right)$, where *f*(*θ*_*i*_) is a function of the within-cohort log(T/R) estimates, *θ*_*i*_. The (*Treat* × *Cohort*) term has an expected MS value of ${\sigma}_e^2+2m{\sigma}_b^2$ and so is the correct error term for testing the treatment effect. PK variability between batches (${\sigma}_b^2$) increases uncertainty in the log(T/R) estimate, and this uncertainty is mitigated (reduced) by increasing the number of observed batches (*c*) just as increasing the number of subjects (individual T/R estimates) mitigates uncertainty due to residual measurement error (${\sigma}_e^2$).

#### Fixed Batch Effect

When (*Treat* × *Cohort*) is handled as a fixed effect, the expression of uncertainty in the log(T/R) estimate considers only residual error; the selected batches are considered to be the only batches of interest to the Test-*vs*-Reference inference. For the case of a single cohort (*c*=1), this reduces to the conventional single-batch implementation of the average bioequivalence methodology.

#### Superbatch

When (*Treat* × *Cohort*) is omitted from the ANOVA model, the residual error variance estimate (${\sigma}_e^2$) is a composite of residual error and the omitted (*Treat* × *Cohort*) term. The model-defined SE of the log(T/R) estimate, still being $\sqrt{MS(Error)/(mc)}$ as in the Fixed Batch Effect approach but now with *df* = *2mc* – 2 (because the model assumes a single cohort), is therefore inflated by an amount that depends on the extent to which variation among batches has increased the “unexplained”, *i.e.*, residual, variability in the data; the expected MS for residual error is ${\sigma}_e^2+\frac{2m{\sigma}_b^2\left(c-1\right)}{\left(2 mc-2\right)\ }$. For the case of a single cohort (*c*=1), this reduces to the conventional single-batch implementation of the average bioequivalence methodology in which the data do not contain information about between-batch PK variability (and therefore there is no inflation of the residual error).

#### Targeted Batch

The Targeted Batch approach implemented here assumes selection of the median *in vitro* batch for the subsequent PK BE study and a perfect correlation between the *in vitro* and PK metrics (*i.e.*, *r* = 1, where *r* is the *in vitro*/*in vivo* correlation coefficient). The SE of the log(T/R) estimate has a minimum value (because of the *r* = 1 assumption) of ${SE}_{min}=\sqrt{\sigma_e^2/m+2{M\sigma}_b^2/1}$, where *M* is a constant related to the sampling distribution of the median of *b* values randomly sampled from a $N\left(0,{\sigma}_b^2\right)$ distribution. That is, ${\sigma}_b^2$ is replaced with the corresponding variance of the sample median ($M{\sigma}_b^2$). If *b* is large and odd, then *M* ≈ *π*/(2(*b* − 1)). For smaller values of *b*, *M* is computed by simulation by repeatedly (100,000 replicates) drawing a random sample of size *b* from the distribution $N\left(0,{\sigma}_b^2\right).$ For *b* = 3, 5, 7, 9, 11, 13, or 15, *M* = 0.44815, 0.28568, 0.20947, 0.16577, 0.13737, 0.11634, or 0.10140, respectively. If *b* is sufficiently large and *r* = 1, *SE* reduces to the conventional value of the standard error for the log(T/R) estimate with a single batch and no batch-to-batch PK variability, namely $\sqrt{\sigma_e^2/m}$.

### Probability of Concluding Bioequivalence

A conclusion of bioequivalence typically requires that the 90% confidence interval on the estimated Test/Reference ratio be fully contained within (0.8000, 1.2500). The probability of this outcome depends on both the model-assumed and true variability of the Test/Reference sampling distribution (Table IV); the former determines the width of the model-determined Test/Reference confidence interval and thus the maximum passable treatment difference (±*k*), the latter determines the probability that a treatment difference within this passable range will be observed given the true underlying variability of the treatment difference sampling distribution. The expressions in Table IV pertain to the bioequivalence outcome of a single PK metric and are detailed in Supplement 2. The probability of concluding bioequivalence used the Student’s *t* distribution parameterized by the model-specific degrees of freedom, with boundary values taken as the model-specific null rejection region divided by the true standard deviation of the treatment difference. Results from simulations across the parameter region were in close agreement.

**Table IV:** Approach-Specific Probability of Concluding Bioequivalence. *μ*_*T*_, *μ*_*R*_, true log-scale test or reference mean; ${\sigma}_b^2$, log-scale within-subject, between-batch PK variance, assumed equal for test and reference; ${\sigma}_e^2$, log-scale within-subject residual error PK variance; *m*, number of subjects per sequence per cohort; *c*, number of cohorts = number of batches per product; *c*=1 in the targeted batch approach which uses a single-cohort PK study design. *M* quantifies variance of the *in vitro* sample median as described earlier. *T*_*df*_ is the centralized Student’s *t*-distribution. A derivation of these entries is provided in Supplement 2

Approach	Maximum treatment difference (*k*) that allows the 90% CI to be contained within [0.8000, 1.2500]	Probability of concluding bioequivalence
Random Batch Effect	$Ln(1.25)-\sqrt{\frac{\sigma_e^2}{mc}+\frac{2{\sigma}_b^2}{c}}\ast {t}_{0.95,c-1}$	$Prob\left\{\frac{-k- Ln\left(\frac{\mu_T}{\mu_R}\right)}{\sqrt{\frac{\sigma_e^2}{mc}+\frac{2{\sigma}_b^2}{c}}}<{T}_{c-1}<\frac{k- Ln\left(\frac{\mu_T}{\mu_R}\right)}{\sqrt{\frac{\sigma_e^2}{mc}+\frac{2{\sigma}_b^2}{c}}}\right\}$
Fixed Batch Effect	$Ln(1.25)-\sqrt{\frac{\sigma_e^2}{mc}}\ast {t}_{0.95,2 mc-c-1}$	$Prob\left\{\frac{-k- Ln\left(\frac{\mu_T}{\mu_R}\right)}{\sqrt{\frac{\sigma_e^2}{mc}+\frac{2{\sigma}_b^2}{c}}}<{T}_{2 mc-c-1}<\frac{k- Ln\left(\frac{\mu_T}{\mu_R}\right)}{\sqrt{\frac{\sigma_e^2}{mc}+\frac{2{\sigma}_b^2}{c}}}\right\}$
Superbatch	$Ln(1.25)-\sqrt{\frac{\sigma_e^2+2{\sigma}_b^2\left(\frac{m\left(c-1\right)}{\left(2 mc-2\right)}\right)}{mc}}\ast {t}_{0.95,2 mc-2}$	$Prob\left\{\frac{-k- Ln\left(\frac{\mu_T}{\mu_R}\right)}{\sqrt{\frac{\sigma_e^2}{mc}+\frac{2{\sigma}_b^2}{c}}}<{T}_{2 mc-2}<\frac{k- Ln\left(\frac{\mu_T}{\mu_R}\right)}{\sqrt{\frac{\sigma_e^2}{mc}+\frac{2{\sigma}_b^2}{c}}}\right\}$
Targeted Batch	$Ln(1.25)-\sqrt{\frac{\sigma_e^2}{m}}\ast {t}_{0.95,2m-2}$	$Prob\left\{\frac{-k- Ln\left(\frac{\mu_T}{\mu_R}\right)}{\sqrt{\frac{\sigma_e^2}{m}+\frac{2{M\sigma}_b^2}{1}}}<{T}_{2m-2}<\frac{k- Ln\left(\frac{\mu_T}{\mu_R}\right)}{\sqrt{\frac{\sigma_e^2}{m}+\frac{2{M\sigma}_b^2}{1}}}\right\}$

### Parameter Ranges

The example PK BE study in this comparative analysis uses 64 subjects; this falls within the range of standard industry practice (9). This study size provides approximately 90% power to demonstrate bioequivalence with a true Test/Reference ratio of 0.89 and log-scale residual error variance (${\sigma}_e^2$) of 0.04 (equivalent to a within-subject coefficient of variation (CV) of 20.2% on the original scale), under the conventional assumption that ${\sigma}_e^2$ is the only variability contributing to the T/R estimate’s standard error. This study size is both realistic for OIDPs and offers convenient flexibility for the current analysis; a 64-subject study can be arranged into 1, 2, 4, 8, 16, or 32 cohorts of 64, 32, 16, 8, 4, or 2 subjects each, respectively (allowing inclusion of 1, 2, 4, 8, 16, or 32 batches each of Test and Reference). If all 64 subjects are arranged into a single cohort (*c*=1), this becomes the conventional single-batch PK BE study design.

Within-subject log-scale residual error variance (${\sigma}_e^2$) is evaluated at a single value of 0.04 (20.2% CV). Within-subject log-scale between-batch PK variance (${\sigma}_b^2$) is evaluated at values of zero, 0.0025 (5% CV), 0.005 (7% CV), 0.01 (10% CV), and 0.02 (14% CV). Of note, ${\sigma}_b^2$ values pertain to variability of a PK metric (Cmax or AUC), not an *in vitro* metric. Between-batch PK variability may in some instances be related to between-batch *in vitro* variability but any quantification of this relationship is beyond the scope of the current work. Between-batch PK variance is assumed to be equal for Test and Reference, although derivation of the analytical solutions is easily adapted to allow the products to differ with respect to between-batch variability. Importantly, all approaches are assessed in the context of existent between-batch PK variability; differences between the approaches pertain to how this feature of the underlying product population is handled in the design and analysis of the PK BE assessment.

## RESULTS

### Single-Batch PK BE Assessment

**Fig. 1:**
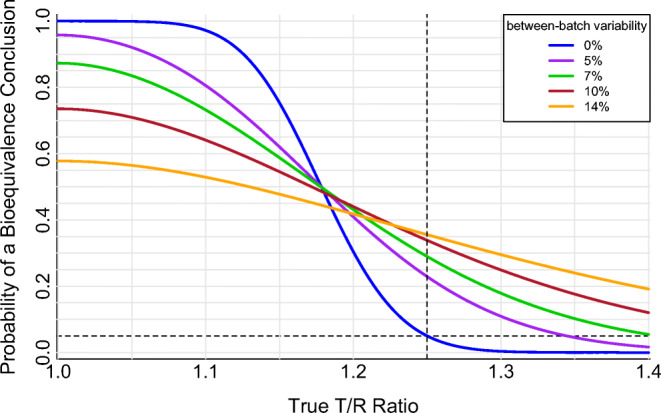
Operating curves of the single-batch two-way crossover pharmacokinetic bioequivalence study in the presence of between-batch pharmacokinetic variability. Two-period, two-sequence (TR, RT) crossover comparing a single randomly selected batch of a test product (“T”) with a single randomly selected batch of a reference product (“R”). Log-scale within-subject residual PK error variance (${\sigma}_e^2$) of 0.04, equal to a within-subject coefficient of variation of 20%. Within-subject between-batch PK variability levels of zero, 5% (${\sigma}_b^2=0.0025$), 7% (${\sigma}_b^2=0.005$), 10% (${\sigma}_b^2=0.01$), or 14% (${\sigma}_b^2=0.02$) are distinguished by color. Sixty-four total clinical study subjects, 32 per treatment sequence. The dashed horizontal line at 0.05 indicates the regulatory expectation of a 5% significance level (*i.e.*, the false-equivalence (type I) error rate, which is the probability of a bioequivalence conclusion when the true T/R ratio is at the 1.25 (or 0.80) bioequivalence limit indicated by the dashed vertical line). Adapted from Benet *et al.* (10)

With zero between-batch PK variability (Fig. 1, blue line), the single-batch two-way crossover PK BE study delivers a high probability of concluding BE for truly equivalent products, a low probability of concluding BE for truly non-equivalent products, and a steep transition in success rate as the true T/R product ratio deviates from 1.00 over the BE window. Importantly, in the absence of between-batch variability, the expected 5% significance level of the bioequivalence test is preserved, *i.e.*, products with a true T/R ratio of 1.25 (or 0.80) will pass the PK BE test on any one PK metric in only 5% of studies.

Even small amounts of between-batch PK variability, however, erode performance of the single-batch two-way PK BE crossover (Fig. 1, Table V). Study power (the probability of correctly concluding BE for truly equivalent treatments) declines, and the type I error rate (the probability of incorrectly concluding BE for truly non-equivalent treatments) rises as between-batch PK variability increases from zero, as previously described (6).

**Table V:** Performance of the Standard Single-Batch Two-way PK BE Crossover for Various Levels of Within-Subject Between-Batch PK Variability. Two-period, two-sequence (TR, RT) crossover comparing a single randomly selected test batch with a single randomly selected reference batch. Log-scale within-subject residual PK error variance (${\sigma}_e^2$) of 0.04, equal to a within-subject coefficient of variation of 20%. Sixty-four clinical study subjects

Between-batch PK variability (log-scale variance, ${\sigma}_b^2$)	Between-batch PK variability (original-scale %CV)	Probability of a BE conclusion for true T/R=1.05 (study power)	Probability of a BE conclusion for true T/R=1.25 (type I error rate)
0	0%	99.9%	5.0%
0.0025	5%	92.1%	22.9%
0.005	7%	83.5%	29.0%
0.01	10%	70.9%	33.8%
0.02	14%	56.5%	35.6%

A common response to low power in PK BE studies is to increase the number of study subjects. This response appropriately addresses residual PK measurement error by increasing the number of PK observations, which reduces the T/R standard error and yields a narrower confidence interval. The consequence of this narrower confidence interval, however, is to further inflate the false equivalence (type I) error rate when between-batch PK variability is present but ignored in the confidence interval construction. Compared to the false equivalence error rates in Table IV for an example 64-subject study, the corresponding false equivalence error rates for a 128-subject study (still with 20% residual error) are 5% (batch variability: 0%), 29.1% (batch variability: 5%), 34.4% (batch variability: 7%), 38.4% (batch variability: 10%), and 39.5% (batch variability: 14%).

The poor performance of the single-batch two-way PK BE crossover study arises from: *(i)* selecting only one batch from a variable population, *i.e.*, using an inadequate batch sample size, and *(ii)* omitting uncertainty due to batch sampling from the T/R confidence interval. The result is a PK BE outcome that is highly dependent on which batch of Test and Reference is selected for the study. The resulting PK BE decision is not directly generalizable beyond the batches used in the study; different batches could yield different bioequivalence outcomes. Overall, the PK BE decision is associated with high error rates for both equivalent (true T/R near 1.00) and non-equivalent products (true T/R near the BE limit, *i.e.*, 1.25 or 0.80).

The multiple-batch approaches that follow address the aforementioned limitations of the single-batch PK BE assessment within a parameter space (number of subjects, number of batches, within-subject residual PK error, within-subject between-batch PK variability) relevant to the OIDP PK BE setting.

### Multiple-Batch PK BE Assessment

#### Increasing Batch Sample Size While Retaining Batch as a Fixed Effect

**Fig. 2:**
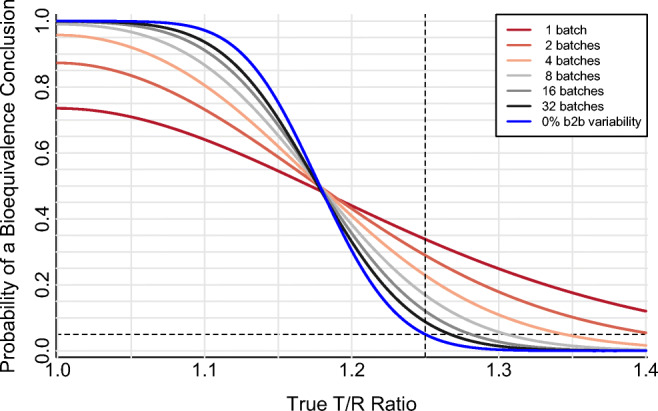
Effect of batch sample size on the performance of the two-way crossover pharmacokinetic bioequivalence study with 10% between-batch variability analyzed using the Fixed Batch Effect approach. Two-period, two-sequence (TR, RT) crossover design with number of batches (equal to number of cohorts, *c*) ranging from one to 32 per product. Log-scale within-subject residual error variance (${\sigma}_e^2$) of 0.04, equal to a 20% within-subject coefficient of variation on the original scale. Log-scale within-subject between-batch PK variance (${\sigma}_b^2$) of 0.01, equal to a 10% within-subject between-batch coefficient of variation on the original scale. Sixty-four total clinical study subjects (N), arranged into *c* cohorts and analyzed per the Fixed Batch Effect approach. The dashed horizontal line at 0.05 indicates the regulatory expectation of a 5% significance level for the statistical bioequivalence test (*i.e.*, false-equivalence (type I) error rate, which is the probability of a bioequivalence conclusion when the true T/R ratio is at the 1.25 (or 0.80) bioequivalence limit indicated by the dashed vertical line). The blue 0% between-batch variability curve is identical to the corresponding blue curve in Fig. 1; this curve illustrates performance of the PK BE test under the conventional assumption of no between-batch PK variability

The Fixed Batch Effect approach (Fig. 2) is a straightforward multiple-batch extension of the conventional single-batch PK BE assessment; the single-batch assessment is simply a special case of the Fixed Batch Effect approach. (The operating curve corresponding to a single batch drawn from a product population with 10% between-batch variability is shown (dark red) in both Fig. 1 (single batch with a range of between-batch variabilities) and Fig. 2 (10% between-batch variability with a range of batch sample sizes)). Inclusion of multiple batches improves the accuracy of the T/R point estimate, and so reduces error rates throughout the operating curve. For example, with 10% between-batch variability in an example two-way crossover with 64 subjects and 20% residual error, the expected probability of failing to identify BE between identical products (true T/R = 1.00) decreases from 26% using a single batch per product (power = 74%) to 4% using four batches per product (power = 96%) with no increase in the number of study participants.

The false equivalence (type 1 error) rate is similarly reduced, but is not restored to the nominal 5% significance level (except for very large batch sample size or negligible between-batch PK variability); the probability of falsely concluding BE for non-equivalent products (true T/R = 1.25 or 0.80) is 34% using a single batch per product and 23% using four batches per product in the example scenario given above and depicted in Fig. 2 (10% between-batch variability, 64 subjects, 20% residual error). Failure to control the false equivalence rate is a direct consequence of a too-narrow T/R ratio confidence interval that omits uncertainty due to batch sampling. The Fixed Batch Effect statistical model assumes that the measured batch, or set of batches, is the complete population of batches of interest for estimating the T/R ratio and so there is no accounting for batches that have not been observed; neither an estimate of batch variability nor batch sample size is included in the Fixed Batch Effect T/R ratio confidence interval. Thus, the Fixed Batch Effect “90%” confidence interval is correct, *i.e.*, provides 90% coverage, only for the specific batches selected; it does not provide the expected coverage for the comparison of the Test and Reference *products*, as has been previously described (6). This is most readily apparent at the BE limit (T/R = 1.25 or 0.80), for which the probability of a BE conclusion is higher than the 5% significance level (*α*) implied by application of a 90% confidence interval (calculated as a (1 − 2*α*) interval by the two one-sided tests bioequivalence procedure (11)).

**Fig. 3:**
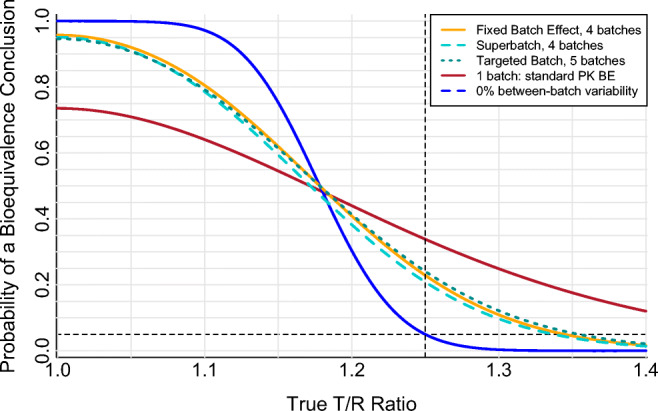
Comparison of multiple-batch two-way crossover pharmacokinetic bioequivalence study approaches for 10% between-batch variability. Four batches (or five, for the Targeted Batch approach) each of a test (“T”) and a reference product (“R”) are compared in a two-period crossover PK BE study. Log-scale within-subject residual error variance (${\sigma}_e^2$) of 0.04, equal to a within-subject coefficient of variation of 20% on the original scale. Log-scale within-subject between-batch PK variance (${\sigma}_b^2$) of 0.01, equal to a within-subject between-batch coefficient of variation of 10% on the original scale. Sixty-four total clinical study subjects (N), arranged into four cohorts (*c*=4) of 16 subjects each with eight subjects per sequence per cohort (*m*=8) for the Fixed Batch Effect and Superbatch approaches, or arranged into a single cohort (*c*=1) of 64 subjects (32 per sequence) for the Targeted Batch approach. The Targeted Batch approach assumes a perfect correlation between the *in vitro* predictor and the *in vivo* PK metric (*r*=1). For comparison, performance of the idealized 0% between-batch variability scenario (N=64, ${\sigma}_e^2$=0.04; blue) and the standard single-batch PK BE approach when between-batch variability is 10% (${\sigma}_b^2$=0.01, N=64, ${\sigma}_e^2$=0.04; red) are included; these curves are identical to the corresponding curves in Figs. 1 and 2. The dashed horizontal line at 0.05 indicates the regulatory expectation of a 5% significance level for the statistical bioequivalence test (*i.e.*, the false equivalence (type I) error rate, which is the probability of a bioequivalence conclusion when the true T/R ratio is at the 1.25 (or 0.80) bioequivalence limit indicated by the dashed vertical line). The orange 4-batch Fixed Batch Effect curve is identical to the corresponding curve in Fig. 2

The Superbatch and Targeted Batch approaches are variations that simplify the statistical model (by omitting batch identity, the Superbatch approach) or the PK BE study (by selecting a single *in vivo* batch from *in vitro* testing of multiple batches, the Targeted Batch approach). Performance of the Fixed Batch Effect, Superbatch, and Targeted Batch approaches is generally similar (Fig. 3) if there is a strong *in vitro*/*in vivo* relationship. The current work characterizes the Targeted Batch approach only for the best-case scenario of a perfect correlation between the *in vitro* predictor and the *in vivo* PK metric (*r* = 1). The effect of a less-than-perfect *in vitro*/*in vivo* relationship on performance of the Targeted Batch approach is detailed elsewhere (12).

The Targeted Batch approach (for *r* = 1) uses the same fundamental idea as the Fixed Batch Effect approach, which is to estimate each product’s typical performance from a sample of multiple batches so that the T/R ratio reflects an estimated average Test response relative to an estimated average Reference response. While the Fixed Batch Effect approach directly averages the PK metrics across multiple batches, the Targeted Batch approach seeks a typical batch using an *in vitro* surrogate for PK. As such, the Targeted Batch approach cannot use the “average” batch — such a batch may not physically exist — and so instead uses the median batch. Thus, for a similar number of batches, the Targeted Batch approach (even with *r* = 1) has poorer T-*vs*-R discrimination (*i.e.*, a less steep operating curve) relative to the Fixed Batch Effect approach (Fig. 3) simply because the sample median is a less efficient estimator of the population average than the sample mean. Performance of the Targeted Batch approach declines further for *r* < 1 (12), and if the *in vitro* median batch is not available for (or has changed prior to) the PK BE study.

The Superbatch approach absorbs within-subject between-batch variability into within-subject residual error, since no term for batch is included in the ANOVA model, and therefore has a wider confidence interval (lower probability of BE success, or power, at any true T/R value) relative to the Fixed Batch Effect approach (Fig. 3). However, under most conditions, the Superbatch confidence interval is only slightly wider than that of the Fixed Batch Effect approach because within-subject between-batch PK variability, now being handled as part of residual error, is considered against *df* driven by the number of T/R observations (which tends to be relatively large, being equal to the number of subjects), not the number of batches. For example, in a 64-subject, 4-cohort (*c*=4, *m*=8), two-way crossover with 20% true residual error (log-scale ${\sigma}_e^2=0.04$) and 10% between-batch PK variability (log-scale ${\sigma}_b^2=0.01$), the apparent residual error in the Superbatch approach increases from the true log-scale value of 0.0400 to only 0.0477, corresponding to an increase in original-scale within-subject CV from the true value of 20.2% to an apparent value of 22.1%. Confidence interval inflation in the Superbatch approach increases as the relative contribution of within-subject between-batch PK variability to overall within-subject variability increases, *i.e.*, with increasing ${\sigma}_b^2/{\sigma}_e^2$ ratio.

Increasing the number of batches improves the performance of all “fixed effect” approaches (Fixed Batch Effect, Superbatch Targeted Batch) relative to the standard single-batch approach by increasing the accuracy of the estimated product geometric mean and, therefore, also of the estimated T/R geometric mean ratio (Table VI). This improvement is substantial for even modest increases in batch sample size. The false equivalence (type I) error rate (evaluated at T/R = 1.25 or 0.80) is similarly improved by increasing batch sample size, although for even relatively large batch sample sizes, type I control is not achieved by the “fixed effect” approaches (Fig. 3, Table VI).

**Table VI:** Performance of Multiple-Batch PK BE Approaches for a Range of Batch Sample Sizes. Two-period, two-sequence (TR, RT) crossover comparing one or more randomly selected test batches with an equal number of randomly selected reference batches. Log-scale within-subject between-batch PK variance (${\sigma}_b^2$) of 0.01, equal to 10% original-scale between-batch PK variability. Log-Scale within-subject residual PK error variance (${\sigma}_e^2$) of 0.04, equal to original-scale PK residual error of 20%. Sixty-four clinical study subjects. To allow selection of the median batch, the targeted batch approach uses an odd number of batches in the *in vitro* screening phase. Italicized rows, which present the performance of a single-batch design, reflect the standard PK be approach; the standard single-batch PK be design is simply a special case of the fixed batch effect, superbatch, and targeted batch approaches

Approach	Number of batches per product	Probability of a BE conclusion for true T/R=1.05 (study power)	Probability of a BE conclusion for true T/R=1.25 (type I error rate)
Fixed Batch Effect	1^*^	*70.9%*	*33.8%*
	2	83.5%	29.0%
	4	92.0%	22.9%
	8	96.7%	16.9%
	16	98.7%	12.1%
Superbatch	1^*^	*70.9%*	*33.8%*
	2	82.5%	27.8%
	4	91.0%	20.9%
	8	95.9%	14.5%
	16	98.3%	9.7%
Targeted Batch (*r* = 1)	1^*^	*70.9%*	*33.8%*
	3	85.1%	28.0%
	5	90.7%	24.1%
	9	95.2%	19.3%
	17	97.9%	14.5%
Random Batch Effect	1	n/a	n/a
	2	0%	0%
	4	26.7%	2.7%
	8	79.5%	4.9%
	16	94.7%	5.0%

^*^The standard PK BE study design compares a single batch of Test to a single batch of Reference, leading to low power and a high rate of decision errors in the presence of between-batch PK variability

#### Increasing Batch Sample Size and Accounting for Random Batch Sampling

**Fig. 4:**
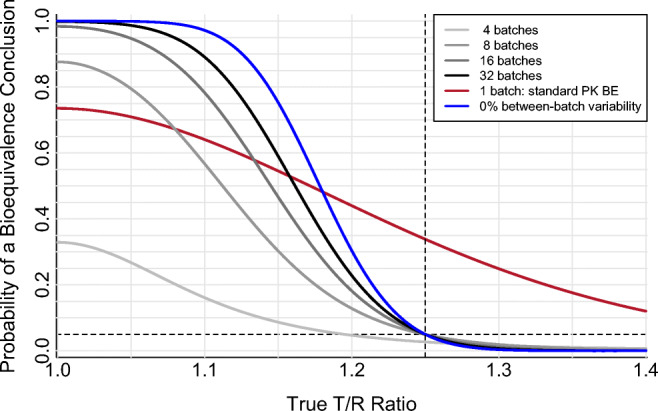
Effect of batch sample size on the performance of the two-way crossover pharmacokinetic bioequivalence study with 10% between-batch variability analyzed using the Random Batch Effect approach. Two-period crossover design with number of batches (equal to number of cohorts, *c*) ranging from four to 32 per product. Log-scale within-subject residual PK error variance (${\sigma}_e^2$) of 0.04, equal to a 20% within-subject coefficient of variation on the original scale. Log-scale within-subject between-batch PK variance (${\sigma}_b^2$) of 0.01, equal to a 10% within-subject between-batch coefficient of variation on the original scale. Sixty-four total clinical study subjects (N), arranged into *c* cohorts and analyzed per the Random Batch Effect approach. For comparison, performance of the idealized 0% between-batch variability scenario (N=64, ${\sigma}_e^2$=0.04; blue) and the standard single-batch PK BE approach when between-batch variability is 10% (${\sigma}_b^2$=0.01, N=64, ${\sigma}_e^2$=0.04; red) are included; these curves are identical to the corresponding curves in Figs. 1, 2, and 3. The dashed horizontal line at 0.05 indicates the regulatory expectation of a 5% significance level for the statistical bioequivalence test (*i.e.*, the false equivalence (type I) error rate, which is the probability of a bioequivalence conclusion when the true T/R ratio is at the 1.25 (or 0.80) bioequivalence limit indicated by the dashed vertical line)

The Random Batch Effect approach restores the PK BE false equivalence (type I) error rate to its expected 5% level by recognizing batch variability in the T/R estimate’s 90% confidence interval. The Random Batch Effect approach estimates within-subject between-batch PK variability separately from residual PK error, and so is able to consider the between-batch component against the batch sample size (here, equal to the number of study cohorts), and the residual error component against the T/R observation sample size (the number of study subjects). With this alignment between the source of variability and its corresponding sample size, the resulting 90% confidence interval demonstrates two key features: (*i*) 90% of intervals contain the true T/R value and (*ii*) ≤5% of intervals lie entirely within the BE region (0.80–1.25) when the true T/R value is 0.80 or 1.25. Thus, the Random Batch Effect approach achieves the expected 5% false positive (type I) error rate (Fig. 4) corresponding to the claimed significance level of the statistical BE test.

However, the Random Batch Effect approach confidence interval is wide (and therefore power is low), for small batch sample sizes due to low *df* on the between-batch variability term; there simply are not enough batches for high confidence (Fig. 4, Table VI). Maximum possible power using four batches per product, achieved with an infinite number of study subjects, reaches only 42.5% for true T/R = 1.05 and 10% between-batch PK variability. The low probability of a BE conclusion for small batch sample sizes (*e.g.*, 4 per product) is also apparent at the BE limit (T/R = 1.25 or 0.80), where the wide distribution of possible observed T/R values allows the tail of this distribution to extend not only into the BE passing region but also past it. For example, with 10% between-batch variability, 64 subjects, and 20% residual error, the observed T/R ratio must fall within 0.964–1.038 in order for the 90% confidence interval around the observed T/R ratio to be contained within 0.80–1.25 using the Random Batch Effect approach. When true T/R=1.25, the left tail of the observed T/R distribution from a two-way crossover is so wide, due to using only 4 batches per product, that it will extend to the left of 0.964. Thus, instead of 5% of the T/R distributional area falling within the BE passing range, only 2.7% does (Fig. 4, Table VI) with the additional 2.3% of the area corresponding to observed T/R values that fail BE because they are too low (despite a true product ratio of 1.25). The Random Batch Effect approach is simply not viable for small batch sample sizes, just as the standard PK BE approach is not viable when the number of subjects is very low.

The Random Batch Effect performance improves substantially with increasing batch sample size (Fig. 4, Table VI), while consistently maintaining type I error rate control.

## DISCUSSION

The IPAC-RS Batch-to-Batch PK Variability Working Group characterized four multiple-batch PK BE design/analysis approaches as extensions of the conventional single-batch approach. None of these alternative approaches requires an increase in the number of clinical study participants. All use multiple batches to improve the accuracy of the T/R point estimate when batches differ with respect to PK response; one (Random Batch Effect) additionally incorporates uncertainty due to batch sampling into the PK BE confidence interval. The three “fixed effect” approaches (Fixed Batch Effect, Superbatch, Targeted Batch) provide higher power to correctly identify true bioequivalence than the standard single-batch approach. The Random Batch Effect approach controls the probability of concluding bioequivalence between non-equivalent products at the expected 5% level.

The multiple-batch PK BE approaches were characterized for batch-to-batch PK variability ranging from 5 to 14% (original-scale CV) to cover a range expected to be commonly encountered for orally inhaled drug products. This range lies below values previously reported in proof of concept PK batch variability studies (4, 6) (approximately 14–23% for AUC, 20–27% for Cmax), and is consistent with the magnitude of between-batch PK variability (9%) used elsewhere for multiple-batch PK BE design simulations (7). Context for between-batch variability magnitude is most readily understood by considering a comparison of identical products (true T/R = 1.00), for which a high PK BE passing rate is expected. For example, a product compared to itself across two different batches is expected to fail approximately one in four adequately powered PK BE comparisons on any one PK metric when the underlying between-batch variability is approximately 10% (Fig. 1).

Sandell and colleagues previously presented the performance of the Superbatch approach as applied to bioequivalent products (specifically, a true T/R ratio ranging from 1.00 to 1.10) (7). The current work extends the Superbatch characterization to T/R > 1.10 (or T/R < 0.91), including to non-equivalent Test and Reference products (T/R ≥ 1.25 or T/R ≤ 0.80). Both the current and prior reports identify a 77% probability of concluding bioequivalence on any one PK metric in a conventional PK BE study when single batches of identical products (true T/R = 1.00) are chosen at random and tested in a two-way crossover of 72 subjects (36 per sequence) with 20% residual error (log-scale ${\sigma}_e^2=0.04$) and 9% between-batch variability (log-scale ${\sigma}_b^2=0.0089$). When the Superbatch approach is implemented in the same scenario using three batches per product instead of one, the probability of concluding bioequivalence on any one PK metric is identified as 90.8% in Sandell *et al*. (7) (calculated as $\sqrt[0.25]{0.68}$), lower than the 94.5% value from the implementation reported here. One known difference between the implementations is that the multiple-cohort design presented here ensures that all batches are equally represented in the PK BE study (thus maximizing the accuracy of the estimated T/R ratio), while the Sandell *et al.* implementation samples at random from Test and Reference “superbatch” pools for each dosing instance.

Performance of the Targeted Batch approach was characterized under the ideal case of a perfect correlation between the predictive *in vitro* test and the PK metric (*r* = 1), availability of the median Test and median Reference batch for the PK BE study, and no change in the relative PK performance of batches between *in vitro* and subsequent *in vivo* testing. These simplifying assumptions were made so that the probability of BE success could be calculated with a numerical solution, without need for simulation. Real-world application of a Targeted Batch approach, including *r* < 1, delivers less sensitivity to product differences (a flatter PK BE operating curve) as the *in vitro* batch selection process becomes less effective at identifying the Test and Reference PK median. This effect is characterized in detail separately (12).

The benefit of multiple batches has long been recognized for OIDP *in vitro* bioequivalence testing; the US-FDA requires “three or more” manufacturing batches per product (13) and the EMA requires “at least three consecutive batches of the test product and three batches of the reference product” (14). Similarly, OIDP PK BE testing is substantially improved with a modest increase in batch sample size, *e.g.*, from one to four (or five, for the Targeted Batch approach), using any of the “fixed effect” approaches (Fixed Batch, Superbatch, Targeted Batch).

In part, the high power when multiple batches are used is attributable to the improved accuracy of the T/R ratio estimate. However, as with the standard single-batch PK BE study, power is artificially high throughout the operating range as a consequence of a too-narrow confidence interval for the “fixed effect” approaches that retain the PK BE convention of excluding uncertainty due to batch sampling from the T/R ratio confidence interval. In these approaches, non-equivalent products have an artificially high likelihood of passing the PK BE test because the too-narrow confidence interval does not admit that the observed “successful” T/R point estimate is associated with more uncertainty than the confidence interval captures. The false equivalence (type I) error rate of the “fixed effect” approaches is roughly fourfold higher than the claimed significance level of the PK BE test for typical study sizes and residual error magnitudes.

When batch is identified by the statistical model as a random effect, the expected 5% false equivalence rate is restored. However, the relatively wider T/R confidence interval generated by the Random Batch approach requires a high number of batches (*e.g.*, ≥ eight per product) to achieve adequate power using the standard [0.8000, 1.2500] bioequivalence limits. Real-world implementation (for which the true magnitude of between-batch PK variability is not known) may additionally struggle to accurately estimate between-batch PK variability from a small batch sample, including for the Reference product for which batch-to-batch PK variability may be apparent only with intermittent sampling of commercially available batches to capture changes in critical process parameters and input ingredients. Accounting for Reference product batch-to-batch variability *via* an expansion of existing Reference-scaled bioequivalence methodology (15) could make the Random Batch Effect approach viable; this interesting adaptation was beyond the scope of the current work.

Thus, between-batch variability is currently a confounding problem for OIDP PK BE testing. Eliminating, or substantially minimizing, between-batch PK variability of OIDPs may not be realistic; the low systemic availability of these locally acting products creates a wide window of opportunity for PK variation. Furthermore, the relationship between critical process parameters and PK response is often complex and poorly understood. Additionally, generic OIDP developers have no control over variability of the marketed Reference product. Handling batch as a random effect in PK BE testing requires either a large number of batches — a potentially undesirable barrier to development — or an expansion of the Reference-scaling methodology to allow this additional source of Reference product variability to influence the BE goalposts. The fundamental premise of handling batch as a fixed effect, namely that the inference on the selected batches can be correctly generalized to the population of all batches, may gain validity with continued identification and understanding of bio-relevant critical quality attributes and critical process parameters, especially if the selected PK BE batches can be shown to represent known product diversity. Regardless of the specific multiple-batch approach used, inclusion of multiple batches reduces PK BE decision error rates relative to the standard single-batch crossover study design.

## CONCLUSION

Evaluation of the standard single-batch and four multiple-batch PK bioequivalence study designs quantitated bioequivalence decision error rates in the presence of PK between-batch variability, a topic of regulatory/industry discussion for over a decade. None of the multiple-batch approaches required an increase in the number of study subjects. Extension of the standard single-batch approach to any “fixed effect” multiple-batch approach substantially increased study power with a modest increase in number of batches per product (*e.g.*, from one to four). The stipulated 5% significance level of the statistical bioequivalence test, however, was maintained only when batch was identified in the statistical model as a random effect. This work offers comparative, quantitative information on PK bioequivalence design/analysis options to mitigate the confounding influence of PK variability among manufacturing batches.

## Supplementary Information

ESM 1 (DOCX 58 kb)
