# Supplementary material for: Performance of Multiple-Batch Approaches to Pharmacokinetic Bioequivalence Testing for Orally Inhaled Drug Products with Batch-to-Batch Variability
Source: AAPS PharmSciTech. 2021 Aug 19;22(7):225. doi: 10.1208/s12249-021-02063-1 (PMC8376725; doi:10.1208/s12249-021-02063-1)
Supplement: Supplementary file 1 — (DOCX 58 kb) [file 12249_2021_2063_MOESM1_ESM.docx]

**Supplement 1**

The statistical model of a multiple-cohort, two-way crossover design can be given as a straightforward extension of the conventional two-way linear model by including terms for cohort, an interaction between cohort and sequence, and an interaction between cohort and treatment:

$$y_{ijkl}=\mu+C_{i}+S_{j}+{(C\times S)}_{ij}+ {\beta_{k}{(C\times S)}_{ij}+P}_{l}+T_{jl}+{(C\times T)}_{ijl}+\varepsilon_{ijkl}$$

$y_{ijkl}$ is the log-scale PK metric (Cmax or AUC) observed in the *l*th period (*l* =1, 2) for the *k*th subject (*k* = 1, 2, ... *m*) of the *j*th sequence group (*j* = 1, 2) in the *i*th cohort (*i* =1, 2, ... *c*); $\mu$ is overall mean response. This model reverts to the standard analysis of variance (ANOVA) model for a two-way crossover design if all terms involving cohort (*C*) are removed and the *i* subscript is dropped.

$C_{i}$, $S_{j}$ and ${(C\times S)}_{ij}$ are the effects for cohort, sequence and the interaction between cohort and sequence; $\beta_{k}{(C\times S)}_{ij}$ is the random effect of subject *k* in sequence group *j* of cohort *i* with $\beta_{k(ij)}$ samples being independent and identically distributed values from a Normal distribution with $\sigma_{p}^{2}$ between-subject variance, *i.e.*, $\beta_{k(ij)} i.i.d.\sim N(0,\sigma_{p}^{2})$. Collectively, these four terms represent the overall effect of subject. Of note, handling between-subject effects as fixed or random is of no consequence to the standard error of the treatment effect (which is a within-subject effect) and is therefore inconsequential to method performance.

$P_{l}$ is the fixed effect of period, and $\varepsilon_{ijkl}$ is within-subject random residual error associated with subject *k* in period *l* of sequence *j* of cohort *i*, $\varepsilon_{ijkl} i.i.d.\sim N(0,\sigma_{e}^{2})$.

$T_{jl}$ is the overall fixed effect of treatment in period $l$ of sequence $j$ (*i.e.*, the overall effect of Test [T] or Reference [R]), and ${(C\times T)}_{ijl}$ is the additional cohort-specific (*i.e.*, batch-specific) effect of treatment for cohort *i* in period $l$ of sequence $j$.

If batch within product is considered as a random effect then, as a consequence of using different batches in each cohort, ${(C\times T)}_{ijl} i.i.d.\sim N(\theta_{jl},\sigma_{b}^{2})$ where $\sigma_{b}^{2}$ is within-subject between-batch variance and $\theta_{jl}=\ln(\mu_{jl})$, *i.e.,* $\theta_{T}=\ln(\mu_{T})$ and $\theta_{R}=\ln(\mu_{R})$. The $T_{jl}$ term then provides the estimate of log (T/R) across cohorts and is tested in the ANOVA with the error term being ${(C\times T)}_{ijl}$ instead of the usual residual error term.

If batch is modeled as a fixed effect, ${(C\times T)}_{ijl}$ is a fixed effect in the ANOVA, and the overall treatment effect, $T_{jl}$, is tested in the ANOVA against the residual error term $\varepsilon_{ijkl}$.

The ${(C\times T)}_{ijl}$ term is included in the Fixed Batch Effect and Random Batch Effect models but excluded from the Superbatch model, and excluded (by definition) from all single-cohort, *i.e.*, single-batch, designs including the Targeted Batch approach by virtue of this (and all ANOVA terms involving cohort) having zero degrees of freedom.

**Supplement 2**

Consider the usual null and alternative hypotheses for PK BE studies:

$$H_{0}:\left| \mu_{T}-\mu_{R} \right|\geq ln(1.25) vs H_{1}: \left| \mu_{T}-\mu_{R} \right|<ln(1.25)$$

where $\mu_{T}$ and $\mu_{R}$ denote true Test and Reference log mean PK parameter values. $H_{0}$ is rejected if the observed treatment difference is small, *i.e.*, if $\left| \hat{\mu}_{T}-\hat{\mu}_{R} \right|<k$.

Thus, for any design:

$k=\ln\left( 1.25 \right)-t_{1-\alpha,df}SE$($\hat{\mu}_{T}-\hat{\mu}_{R})$

where $SE$($\hat{\mu}_{T}-\hat{\mu}_{R})$ is the standard error of the estimated treatment difference.

Across many repeated PK BE studies, each contributing one $\hat{\mu}_{T}-\hat{\mu}_{R}$ estimate, $SE$($\hat{\mu}_{T}-\hat{\mu}_{R})$ could be estimated empirically as the standard deviation of the resulting distribution of log treatment differences. If the batch sample for each study is drawn at random from the product population, the expected value of $SE$($\hat{\mu}_{T}-\hat{\mu}_{R})$ will equal the value of the Random Batch Effect approach (**Table III**) for normally distributed data. However, in practice only a single study is run, with a single sample of batches. In a single study, $SE$($\hat{\mu}_{T}-\hat{\mu}_{R})$ is estimated via the fitted ANOVA model. The maximum treatment difference (±*k*) that allows a BE conclusion depends on the model-specific estimate of $SE$($\hat{\mu}_{T}-\hat{\mu}_{R})$ and the associated critical *t* value ($t_{1-0.05,df}$). Denoting these approach-specific (Fixed Batch Effect, Superbatch, Targeted Batch or Random Batch Effect; **Table III**) values as $\tilde{SE}$ and $\tilde{df}$, the ‘passing window’ (±*k*) within which the observed treatment difference must fall so that the associated $100\left( 1-2\alpha\right)\%$ confidence interval lies within the BE acceptance limits (0.8000, 1.2500) is given as:

$k=\ln\left( 1.25 \right)-t_{1-0.05,\tilde{df}}\tilde{SE}$($\hat{\mu}_{T}-\hat{\mu}_{R})$.

By extension, for any true value *Δ* of $\mu_{T}-\mu_{R}$, the probability of concluding BE can be expressed as:

$Pr(BE)=Pr(\left| \hat{\mu}_{T}-\hat{\mu}_{R} \right|<\left. k \right|\mu_{T}-\mu_{R}=\Delta)$, which can be expressed as

$$Pr\left( BE \right)=Pr\left( \frac{-k-\Delta}{\breve{SE}(\hat{\mu}_{T}-\hat{\mu}_{R})}<T_{\tilde{df}}<\frac{k-\Delta}{\breve{SE}(\hat{\mu}_{T}-\hat{\mu}_{R})} \right)$$

where $T_{df}$ is the centralized Student’s *t*-distribution and $\breve{SE}$ denotes the standard deviation of the true distribution of observed treatment difference values when the observed batches are only a selection from a larger population of batches, *i.e.*, when batch is random, $\breve{SE}\left( \hat{\mu}_{T}-\hat{\mu}_{R} \right)=$ $\sqrt{\frac{\sigma_{e}^{2}}{mc}+\frac{2\sigma_{b}^{2}}{c}}$. That is, the probability of concluding BE is the probability that the observed treatment difference will be not greater than ±*k* given the true underlying variability of the $\hat{\mu}_{T}-\hat{\mu}_{R}$ sampling distribution.
